# Impact of TCM on Tumor-Infiltrating Myeloid Precursors in the Tumor Microenvironment

**DOI:** 10.3389/fcell.2021.635122

**Published:** 2021-03-04

**Authors:** Jinlong Liu, Yuchen Wang, Zhidong Qiu, Guangfu Lv, Xiaowei Huang, He Lin, Zhe Lin, Peng Qu

**Affiliations:** ^1^Department of Pharmacy, Changchun University of Chinese Medicine, Changchun, China; ^2^Center for Cancer Research, National Cancer Institute, Frederick, MD, United States

**Keywords:** traditional Chinese medicine, regulatory mechanism, tumor microenvironment, tumor-infiltrating myeloid precursors, cancer immunotherapy

## Abstract

The tumor microenvironment (TME) is composed of tumor cells, blood/lymphatic vessels, the tumor stroma, and tumor-infiltrating myeloid precursors (TIMPs) as a sophisticated pathological system to provide the survival environment for tumor cells and facilitate tumor metastasis. In TME, TIMPs, mainly including tumor-associated macrophage (TAM), tumor-associated dendritic cells (DCs), and myeloid-derived suppressor cells (MDSCs), play important roles in repressing the antitumor activity of T cell or other immune cells. Therefore, targeting those cells would be one novel efficient method to retard cancer progression. Numerous studies have shown that traditional Chinese medicine (TCM) has made extensive research in tumor immunotherapy. In the review, we demonstrate that Chinese herbal medicine (CHM) and its components induce tumor cell apoptosis, directly inhibiting tumor growth and invasion. Further, we discuss that TCM regulates TME to promote effective antitumor immune response, downregulates the numbers and function of TAMs/MDSCs, and enhances the antigen presentation ability of mature DCs. We also review the therapeutic effects of TCM herbs and their ingredients on TIMPs in TME and systemically analyze the regulatory mechanisms of TCM on those cells to have a deeper understanding of TCM in tumor immunotherapy. Those investigations on TCM may provide novel ideas for cancer treatment.

## Introduction

Malignant tumors have progressively become an important disease of human death (Global Burden of Disease Cancer Collaboration et al., 2017; Bray et al., 2018). Even though the main treatments for malignant tumors are still radiotherapy, surgery, and chemotherapy, traditional Chinese medicine (TCM) has been applied to treat the patients with cancer in China for many years (Qi et al., 2015). TCM can protect cancer patients from complications, increase sensitivity or reduce side effects of conventional treatments, and improve quality of life and survival (Liang et al., 2014; Wang et al., 2020b). According to the theory of TCM, the key reasons for tumor occurrence and development are both the imbalance of Yin/Yang and the prosperous deficiency and evil (Xiang et al., 2019). This is consistent with the basic treatment ideas of modern tumor immunotherapy, which breaks immune tolerance and reverses immune escape to rebuild the body’s normal immune function and enhance antitumor ability. Many scientists believe that CHM reduces tumor growth and metastasis by enhancing antitumor immunity. Some reports have described the application of CHM in cancer treatment (Hsiao and Liu, 2010; Hu et al., 2013; Ting et al., 2015; Wang Z. X. et al., 2018). CHM and their active ingredients restrain the growth of cancer cells directly and prevent the invasion and metastasis of cancer cells by acting on the tumor microenvironment (TME), therefore playing an increasingly important role in antitumor treatment (Lin et al., 2017; Shi et al., 2020). The direct roles of TCM on tumor cells have been discussed in many articles (Qiu and Jai, 2014; Fan et al., 2020); in the review, we focus on the impact of TCM on TME, especially for immune cells within TME. The TME is a complicated pathological system, which is involved in the interaction of plenty numbers of cells such as tumor cells, lymphatic/blood endothelial cells, and tumor-infiltrating myeloid precursors (TIMPs) to provide the survival environment for tumor cells and facilitate tumor metastasis. A majority of those TIMPs are myeloid-derived hematopoietic cells, including tumor-associated macrophages (TAMs), dendritic cells (DCs) in tumor, and myeloid-derived suppressor cells (MDSCs). Under physiological conditions, multipotent hematopoietic stem cells are differentiated into immature myeloid cells (IMCs) that develop into mature myeloid cells with multiple functions (Qu et al., 2016). The three major groups of terminally differentiated myeloid cells—macrophages, DCs, and granulocytes—are essential for the physiological functions of the immune system. These cells protect organisms from pathogens, eliminate necrotic cells, and mediate tissue remodeling through the immune response. However, within the TME with the characteristics of hypoxia, acidity, and interstitial high pressure, those myeloid cells are converted into potent immunosuppressive populations that accelerated the growth, invasion, and metastasis of tumor. Expansion and function of TIMPs within the TME have been investigated in our laboratory and other institutes (Hosseini et al., 2020). However, little is known about the impact of TCM herbs and its active components on those TIMPs in TME. In the review, we summarized the inhibitory roles of TCM herbs and its active components on the growth, invasion, and metastasis of tumor, and focused on their regulatory function on TIMPs in TME, providing novel therapeutic methods for cancer treatment.

## General View for CHM and Their Active Ingredients

Traditional Chinese medicine, as one unique system of medical care, has been used in China and Asia for thousands of years. It is very different from Western medicine and uses a combination of various practices including acupuncture, massage therapy, moxibustion, and herbal remedies. According to the theory of TCM, the occurrence of illness is due to the disturbance of two opposing forces of energy, Yin and Yang. To alleviate symptoms of disease, TCM aims to restore the harmony of Yin and Yang. In recent decades, increasing numbers of patients have been attracted to use TCM as an adjuvant therapy option for various diseases (Jiang et al., 2010). In particular, TCM-based Chinese herbal medicine (CHM) has been shown to exhibit potential therapeutic effects as an adjunctive treatment following surgery, chemotherapy, radiotherapy, or other types of therapy for cancer patients worldwide (Nie et al., 2016). CHM and their compounds have the advantage of availability, efficacy, and relatively low toxicity, compared with other therapy methods. Evidence has confirmed that CHM in combination with chemotherapy or radiotherapy is capable of promoting the efficacy of chemotherapy or radiotherapy and diminishing the limitations and drawbacks induced by them (Qi et al., 2015). The objective of this review is to contribute to a clearer understanding of CHM and active compounds as an adjuvant therapy for cancer, and illustrate the underlying mechanisms of TCM-based CHM on cancer therapy from the point of view of TME.

## Differentiation, Phenotypes, and Function of TIMPs

Tumor-infiltrating myeloid precursors mainly include TAM, tumor-associated DCs, and MDSCs. A large number of studies have shown that TAMs are typical pre-tumor macrophages (M2), which are responsible for the release of immunosuppressive cytokines, chemokines, and growth factors, such as arginase, vascular endothelial growth, and other factors, rendering tumor-specific cytotoxic T lymphocytes hyporesponsive and promoting tumor angiogenesis (Pollard, 2004).

Dendritic cells arise from Lin-CD34+ hematopoietic stem cells and are classified into two different developmental stages: immature DCs (iDCs) in peripheral tissues primarily with the specialized functions of antigen uptake and processing and mature DCs (mDCs) in lymphoid organs with the interaction with antigen-specific T cells. mDCs, the most powerful antigen-presenting cells (APCs), are considered as critical regulators of adaptive immune responses. They can present tumor-associated antigen to T cells and initiate antitumor response. However, in TME, the complex interplay of stromal, immune, and tumor cells leads to DC dysfunction, even becoming immunosuppressive cells. DCs in the TME promote the differentiation of T cells into Treg subtypes, further weaken the antitumor activity mediated by T cells, support the formation of new blood vessels, block antitumor immunity, and stimulate the growth and spread of cancer cells (Ma et al., 2012).

Myeloid-derived suppressor cells originate from bone marrow and are composed of bone marrow progenitor cells and IMCs. In mice, according to their epitope-specific antibodies, they are divided into two subgroups: monocyte CD11b^+^LY6G^–^LY6C^hi^ phenotype (M-MDSCs) and granulocyte CD11b^+^LY6G^+^ LY6C^low^ phenotype (G-MDSCs). Both MDSCs utilize different suppressive mechanisms (Qu et al., 2012). M-MDSCs produce very little reactive oxygen species (ROS) but produce a high level of nitric oxide (NO) and consist of IMCs with the ability to differentiate into macrophages and DCs. This subset of MDSCs mediates immune suppression through the production of NO and arginase. In contrast, G-MDSCs express a high level of ROS and very little NO and are the majority population of MDSCs in tumor-bearing mice. Suppression by G-MDSCs is mediated via ROS and H_2_O_2_. In humans, MDSCs in cancer patients are defined by the combinations of functional markers, such as CD14, CD33, CD11b, and CD66b (Bronte, 2009; Qu et al., 2012; Youn et al., 2012). In different cancer patients, there are different types of MDSCs with suppressive roles (Qu et al., 2016). Therefore, those TIMPs suppress antitumor immune response through different mechanisms within the TME.

## Impact of CHM and Their Active Ingredients on Tumor Growth, Invasion, and Metastasis

### Regulation of CHM and Their Active Ingredients on Tumor Growth

Cancer cells grow wildly and malignantly due to the unlimited proliferation of tumor cells and the mitigation of their apoptosis (Xu et al., 2016). The inhibition roles of CHM on cancer cell growth have been studied broadly for many years, and active components of CHM have been applied for clinical trials (Ling et al., 2014). For instance, kaempferol was identified to repress the mitochondrial biogenesis and antagonize the activity of ERRa and ERRg to impede tumor growth (Wang et al., 2013) (Table 1). In addition, Wang et al. (2011) found that *Spatholobus suberectus* Dunn (SSD) also retarded cancer growth, but its inhibitory mechanism was different from those of kaempferol. SSD inhibits tumor cell growth by inducing mitochondrial apoptosis and inhibiting the cell cycle in the G2/M phase. SSD also increases the inhibition rate of docetaxel and diminishes its side effects (Table 1). Recently, we reported that ginsenosides Rg3, Rg5, Rh2, and CK downregulated the expression of cell division cycle proteins cyclinB1, CDC2, Cytc-B, CDK-4, and CDK-6 to induce tumor cell cycle arrest in the G0/G1 phase (Chen et al., 2018).

**TABLE 1:** Regulatory mechanism of CHM on the growth of tumor.

TCM herbs and their components	Cell lines/related mouse models	Mechanisms	References
**KA**	A549 lung cancer	To exert its anticancer effect by antagonizing ERRs activity	Wang et al. (2013)
**SSD**	MCF-7/HT-29/MCF-10A MCF-7/HT-29-induced colon cancer model	To inhibit cancer cell growth by inducing apoptosis and arresting cell cycle at G2/M checkpoint	Wang et al. (2011)

*KA, kaempferol; SSD, *Spatholobus suberectus* Dunn.*

### Impact on Tumor Invasion and Metastasis

Tumor metastasis is regarded as a major obstacle to successful cancer therapy. The blockage treatments of metastasis provide more survival opportunities for cancer patients (Ma et al., 2020). Recent investigations about the regulation of tumor metastasis were involved in one family of enzymes, the matrix metalloproteinase (MMP) family, which exacerbated tumor metastasis in TME (Kessenbrock et al., 2010). Those data were consistent with our previous findings (Qu et al., 2009, 2011). Thus, the inhibition of CHM and their active ingredients on the activity of MMPs may attenuate tumor migration/metastasis. *Prunella vulgaris* L. (PVL) exhibited capacity to diminish the expression levels of MMP-2 and MMP-9, further reducing liver cancer metastasis (Kim et al., 2012) (Table 2). Chen et al. (2013) found that baicalein isolated from *Scutellaria baicalensis* Georgi (BCL) decreased the levels of MMP-2, MMP-9, and u-PA while elevating the expression of TIMP-1 and TIMP-2 to reduce the migration and metastasis of liver cancer cells through the decreased phosphorylation levels of MEK1 and ERK. In addition, the lung metastasis rate was found to be significantly decreased in the baicalein-treated nude mouse model LCID20 (Chen et al., 2013) (Table 2). As baicalein, formononetin was also found to induce the decreased levels of both MMP-2 and MMP-9 to prevent the lung metastasis of MDA-MB-231 and 4T1 breast cancer cells. However, its role is regulated through PI3K/AKT signaling pathways (Zhou et al., 2014) (Table 2). For human ovarian cancer cell lines, both crude polysaccharides isolated from *Rosa roxburghii* Tratt and tanshinone IIA reduced the high MMP9 expression, which was related to tumor stage and lymph node metastasis (Chen et al., 2014; Zhang et al., 2016) (Table 2).

**TABLE 2:** Functional mechanism of CHM on the invasion and metastasis of tumor.

TCM herbs and their components	Cell lines/related mouse models	Mechanisms	References
**PVL**	HepG2/Huh-7/Hep3B	To suppress cell invasion and migration in liver cancer cells by attenuating MMPs	Kim et al. (2012)
**BCL**	HCC/MHCC97H MHCC97H-induced liver cancer model	To inhibit the invasion and metastatic capabilities of cancer cells via the downregulation of ERK pathway	Chen et al. (2013)
**FMT**	MDA-MB-231 cells MDA-MB-231-induced breast cancer model	To suppress MMP-2 and MMP-9 to inhibit migration and invasion of breast cancer cells through PI3K/AKT signaling pathways	Zhou et al. (2014)
**CP**	A2780	To decrease MMP-9 expression	Chen et al. (2014)
**TanIIA**	SW480	To reduce the level of vimentin and MMP-9, and enhance the expression levels of E-cadherin	Zhang et al. (2016)
**UA**	HCT116/HCT-8-induced colorectal cancer models	To suppress the invasive potential of cancer cells by regulating the TGF-beta1/ZEB1/miR-200c signaling pathway	Zhang Y. et al. (2019)
**CBS**	HeLa and HeLa-induced cervical cancer model	To downregulate MAPK/TGF-β/Nrf2 signaling pathways	Peng et al. (2020)
**QYHJ**	BxPC3/SW1990HM SW1990HM-induced pancreatic cancer model	To reduce the levels of vimentin, N-cadherin and Slug, increase the expression level of E-cadherin	Zhang et al. (2013)

*BCL, baicalein; CBS, *Conyza blinii* saponin; CP, crude polysaccharides from *Rosa roxburghii* Tratt; FMT, formononetin; MMPs, matrix metalloproteinases; PVL, *Prunella vulgaris* L.; QYHJ, Qing-Yi-Hua-Ji formula; TanII A, tanshinone IIA; UA, ursolic acid.*

Either cancer cells or stroma cells activate transforming growth factor-beta (TGF-β) to produce MMPs or other factors in the extracellular matrix, further facilitating the tumor metastasis (Stuelten et al., 2005). TCM herbs such as ursolic acid (UA) treatment reduces the expression levels of TGF-β1 and the phosphorylation of Smad2/3 to block Zinc Finger E-Box Binding Homeobox 1 (ZEB1), further inducing the increased levels of miR-200c to reduce the invasive potential of colon cancer cells, suggesting that UA prevented colon cancer cell invasion through the TGF-β1/ZEB1/miR-200c signaling pathway (Zhang L. et al., 2019) (Table 2). *Conyza blinii* saponin (CBS) isolated from *Eschenbachia blinii* (H.Lév.) Brouillet inhibits the activation of TGF-β signaling pathway and the phosphorylation of ERK, JNK, and p38 MAPK. CBS also reduces the expression of Nrf2 in HeLa cells, inhibits the activation of ARE, and increases the level of ROS (Peng et al., 2020) (Table 2).

Epithelial–mesenchymal transition (EMT) is also shown to promote tumor metastasis. Some CHM inhibit EMT to prevent tumor metastasis. Qingyihuaji formula (QYHJ) impaired EMT in pancreatic cancer to restrain tumor metastasis via the decreased levels of vimentin, N-cadherin, and Slug (Zhang et al., 2013) (Table 2). Therefore, CHM and their active constituents inhibited the growth, invasion, and metastasis of different types of tumor through the blockage of tumor-related signaling pathways.

## The Impact of CHM and Their Active Ingredients on TIMPs in TME

Tumor microenvironment is quite different from the physiological characteristics of normal tissues at the cellular and tissue levels. As a sophisticated pathological system, TME is involved in the crosstalk between tumor cells and TIMPs to provide the nourishment for tumor cells, improving the survival environment for tumor cells, and accelerates tumor metastasis (Sun, 2015; Kumar et al., 2016). There is increasing evidence that CHM mediates the TME through downregulating the suppressive function of TIMPs, including TAMs, DCs in tumor, and MDSCs (Guo et al., 2015).

### The Regulatory Roles on TAMs

Macrophages, one type of versatile immunocytes, display different phenotypes, depending on their microenvironment. Activated macrophages are classified into the M1 and M2 phenotype. In general, M1 macrophages foster inflammation response against invading pathogens and tumor cells, whereas M2 macrophages tend to exert an immune suppressive phenotype, favoring tumor progression (He et al., 2020a). Even though TAMs exhibit either polarization phenotype, they are considered as M2-like phenotype-acquired macrophages and produce epidermal growth factor (EGF) and MMPs to accelerate the migration and angiogenesis of tumor in TME (Guo et al., 2018; He et al., 2020b). Therefore, therapeutic strategies are to re-educate the M2 phenotype (pro-tumorigenesis) into antitumor M1 phenotype (anti-tumorigenesis), preventing the promotion roles of TAM in tumors (Quail and Joyce, 2013).

#### Murine Cancer Cell Lines/Models

**FIGURE 1:**
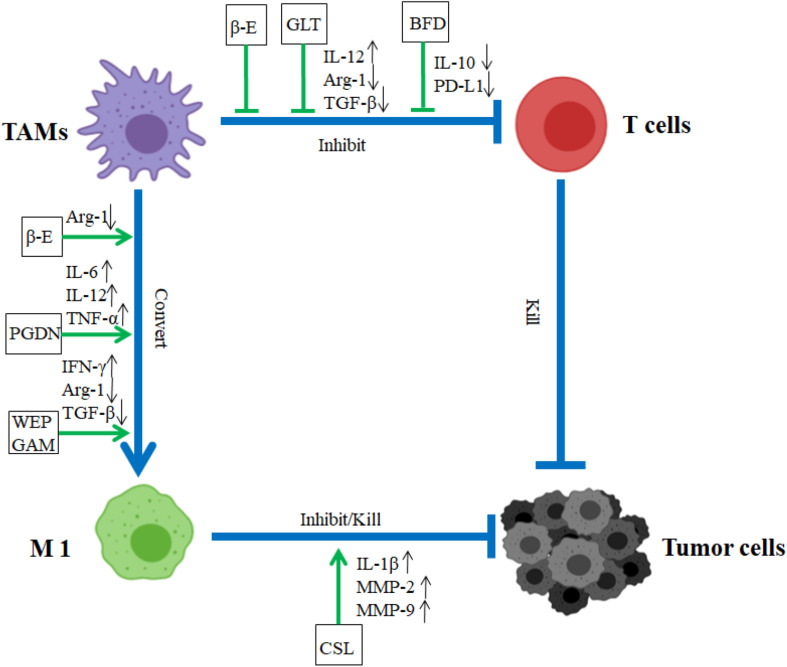
Impact of CHM on TAMs in TME. In TME, TAMs accelerated tumor migration and invasion. CHM downregulated the roles of TAM and promoted the transformation of TAMs from M2 type to M1 type. In TME, β-E, GLT, and BFD blocked the inhibition function of TAM. β-E, PGDN, and WEPGAM triggered TAM conversion from M2 to M1 type, CSL retarded tumor growth. BFD, Bu Fei decoction; β-E, β-elemene; CSL, *Crocus sativus* L; WEPGAM, water extract of *Panax ginseng* C. A. Mey. and *Astragalus mongholicus* Bunge; PGDN, *Panax ginseng* C. A. Mey.-derived nanoparticles; GLT, *Garcinia livingstonei* T. Anderson; blue lines demonstrated the promotion (→) or inhibition (⊣) roles among immune cells (TAM or T cells) and tumor cells. Green lines indicated the promotion (→) or inhibition (⊣) roles of CHM.

Some TCM herbs were found to convert TAMs (M2-like phenotype) to the M1-like phenotype and block the promotion functions of TAMs on tumor. Water extract of *Panax ginseng* C. A. Mey. and *Astragalus mongholicus* Bunge (WEPGAM) treatment can remarkably inhibit the transplanted tumor growth in mice (Chen et al., 2019) (Figure 1 and Table 3). In addition, the reprogramming of TAMs toward M1-like macrophages is also regulated by TCM active components such as β-elemene (βe), which reduces the expression of Vimentin, N-cadherin, and Arg-1, and upregulates the expression of E-cadherin and iNOS to regulate the poles of macrophages from M2 to M1, inhibiting the proliferation, migration, and invasion of lung cancer cells (Yu et al., 2017) (Figure 1 and Table 3). Our previous data demonstrated that some of Ginsenosides isolated from *Panax ginseng* C. A. Mey. were able to convert TAM polarization from M2-like to M1-like to attenuate tumor metastasis (Zhang Y. et al., 2019). Recently, *Panax ginseng* C. A. Mey.-derived nanoparticles (PGDN) were also found to have similar regulatory roles on TAMs in melanoma. Cao M. et al. (2019) found that PGDN significantly reduced the level of CD206 in M2-like macrophages and upgraded the expression of CD80, CD86, MHC-II, and TLR2/4 to induce the increased numbers of M1 macrophages, reducing tumor growth in vaccinated mice and human melanoma cells (Figure 1 and Table 4).

**TABLE 3:** Effect of CHM on TIMPs *in vitro*.

TCM herbs and their components	Murine tumor cell line	TIMPs	Mechanisms	References
**WEPGAM**	LLC lung cancer	TAMs	To promote the transformation of M2 phenotype to M1 phenotype	Chen et al. (2019)
**βE**	LLC lung cancer	TAMs	To skew TAMs polarity toward the M1 phenotype	Yu et al. (2017)
**GIBISL**	4T1 breast cancer	TAMs	To reverse M2 phenotype macrophage polarization	Wang et al. (2015)
**LBP**	CT26 colon cancer	DCs	To prompt DC maturation	Wang Z. X. et al. (2018)
**GLPS**	P815 mastocytoma	DCs	To activate specific CTL through increasing IFNγ production to stimulate DC maturation	Cao and Lin (2002)

*DC, dendritic cells; GIBISL, total flavonoid from *Glycyrrhiza inflata* Batalin and (its active ingredient) isoliquiritigenin; GLPS, *Ganoderma lucidum* polysaccharides; LBP, *Lycium barbarum* L. polysaccharide; MDSCs, myeloid-derived suppressor cells; TADC, decoction tumor-associated dendritic cells; TAM, tumor-associated macrophage; WEPGAM, water extract of *Panax ginseng* C. A. Mey. and *Astragalus mongholicus* Bunge; βE, β-elemene.*

**TABLE 4:** Regulation of CHM on TIMPs in TME of murine tumor models.

TCM herbs and their components	Murine cancer xenograft models	TIMPs	Mechanisms	References
**PGDN**	B16F10 cells induced melanoma	TAMs	To skew TAMs polarity toward the M1 phenotype	Cao Y. et al. (2019)
**GLT**	AOM/DSS-induced colorectal cancer model	TAMs	To reduce TAM production	Sui et al. (2020)
**RSV**	LLC lung cancer model	TAMs	To diminish tumor-associated M2 polarized macrophages	Sun et al. (2017)
**DNC**	4T1 breast cancer model.	TAMs	To diminish tumor-associated M2 polarized macrophages	Shiri et al. (2015)
**QRHX**	LLC lung cancer model	TAMs	To decrease TAM production	Xu et al. (2017)
**YPF**	LLC-Luc-induced lung cancer model	TAMs	To promote the transformation of M2 phenotype to M1 phenotype	Wang Y. et al. (2019)
**TR**	4T1 breast cancer model/AOM/DSS-induced colorectal cancer model	TAMs	To skew TAMs polarity toward the M1 phenotype	Li et al. (2020)
**CT**	JC-induced breast cancer model	DCs	To activate DCs to present antigens to T cell	Chang et al. (2011)
**PPS**	TC-1 cervical cancer model	TADCs	To reverse the immature state of TADCs	Wang Y. et al. (2019)
**PAMB PCPP**	4T1 breast cancer model	DCs	To improve DC immune activity	Chang et al. (2015)
**SBS**	AOM/DSS-induced colorectal cancer model	MDSCs	To block the immunosuppressive activity of MDSCs	Lin et al. (2015)
**SGJP**	4T1 breast cancer model	MDSCs	To block the immunosuppressive activity of MDSCs	Lu et al. (2017)
**PA**	4T1 breast cancer model	MDSCs	To decrease the number of MDSCs	Zheng et al. (2018)
**RSV**	LLC lung cancer model	MDSCs	To inhibit the function of MDSCs	Zhao Y. et al. (2018)
**AP, AS, CS, SD**	4T1 breast cancer model	MDSCs	To diminish the number of Tregs and MDSCs	Yue et al. (2018)
**KRG**	EL-4 thymoma model	MDSCs	To disrupt the function of MDSCs	Jeon et al. (2011)
**JHD**	H_22_ hepatoma carcinoma model	MDSCs	To inhibit immunosuppressive activity of MDSCs	Xie et al. (2020)
**ART**	4T1 breast cancer model	MDSCs	To impair the activity of Tregs and MDSCs	Cao Y. et al. (2019)
**YHD**	4T1 breast cancer model	MDSCs	To inhibit the activity of MDSCs	Mao et al. (2018)
**ICA**	4T1 breast cancer model	MDSCs	To impair the suppressive activity of MDSCs	Zhou et al. (2011)
**CA**	LM85 osteosarcoma model.	MDSCs	To block the function of MDSCs	Horlad et al. (2013)
**BYJD**	4T1 breast cancer model	MDSCs	To decrease the number of MDSC	Tian et al. (2020)

*AP, *Andrographis paniculata* (Burm.f.) Nees; AS, *Eleutherococcus senticosus* (Rupr. & Maxim.) Maxim.; CS, *Camellia sinensis* (L.) Kuntze; SD, *Scleromitrion diffusum* (Willd.) R.J. Wang; ART, artemisinin; BYJD, Bao-Yuan-Jie-Du decoction; CA, corosolic acid; CT, *Carthamus tinctorius* L.; DC, dendritic cells; DNC, dendrosomal curcumin; GLT, *Garcinia livingstonei* T. Anderson; ICA, icariin from *Epimedium sagittatum* (Siebold & Zucc.) Maxim.; JHD, Jianpi Huayu decoction; KRG, Korean red ginseng; MDSCs, myeloid-derived suppressor cells; PA, water extract of pilose antler; PAMB or PCPP, polysaccharide purified from *Astragalus mongholicus* Bunge or *Codonopsis pilosula* subsp. *pilosula*; PGDN, *Panax ginseng* C. A. Mey.-derived nanoparticles; PPS, *Pinellia pedatisecta* Schott; QRHX, Qing-Re-Huo-Xue formulae; RSV, resveratrol; SBS, Shen-ling-Bai-zhu San; SGJP, Shu-Gan-Jian-Pi formula; TADC, decoction tumor-associated dendritic cells; TAM, tumor-associated macrophage; TR, triptolide; YHD, Yang-He decoction; YPF, Yu-Ping-Feng.*

**FIGURE 2:**
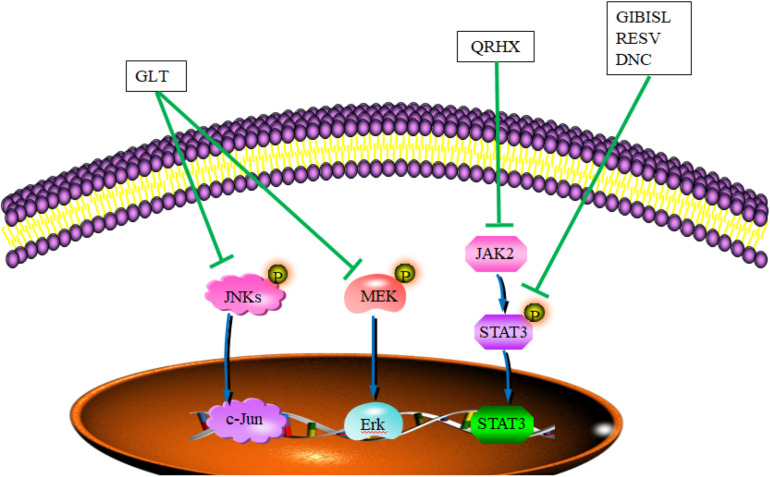
CHM and their active ingredients skewed TAM polarity toward the M1 phenotype through signaling pathways. In TME, GLT blocked M2 macrophage polarization in colitis-associated tumorigenesis through downregulating JNK and ERK signaling. QRHX impaired the function of TAM and impeded tumor growth in tumor-bearing mice through reduced phosphorylation levels of JAK2/STAT3. RESV, DNC, and GIBISL skewed the polarization of TAM toward M1 through the inactivation of STAT3. DNC, dendritic curcumin; GLT, *Garcinia livingstonei* T. Anderson; QRHX, Qing-Re-Huo-Xue formulae; RESV, resveratrol; GIBISL, total flavonoid from *Glycyrrhiza inflata* Batalin and (its active ingredient) isoliquiritigenin. Blue lines demonstrated the promotion (→) or inhibition (⊣) roles of signal path. Green lines indicated the promotion (→) or inhibition (⊣) roles of CHM.

Chinese herbal medicine and their active components exhibit blockage ability to the roles of TAMs in TME through JAK/STAT, JNK, and ERK signaling pathway, which is involved in mediating the growth, invasion, and metastasis of tumor (Lin et al., 2019). Total flavonoid from *Glycyrrhiza inflata* Batalin (GIB) and its important ingredient, isoliquiritigenin (ISL), reverse the polarization of M2 phenotype macrophages to retard tumor invasion through inhibiting the gene and protein expression of Arg-1. In addition, both GIB and ISL upregulate protein expression of iNOS, enhance the expression of microRNA 155 and its target gene SHIP1, and downregulate the phosphorylation of STAT3 and STAT6 (Wang et al., 2015) (Figure 2 and Table 3). *Garcinia livingstonei* T. Anderson (GLT) elevates the expression level of iNOS and IL-12, and reduces the expression levels of IL-6, TNF-α, Arg-1, and IL-1β on TAMs to impede the tumor progression through the inhibition of STAT3, JNK, and ERK signaling pathway (Sui et al., 2020) (Figure 2 and Table 4). Both Resveratrol (RSV) and Dendrosomal Curcumin (DNC) are revealed to downregulate the expression levels of IL-10 and Arg1 on TAM through the inactivation of STAT3 to reduce the numbers of TAM, further inhibiting tumor growth and metastasis (Shiri et al., 2015; Sun et al., 2017) (Table 4). The transcription factors STAT3 and STAT1 appear to play opposite roles in tumorigenesis. STAT3 activation has been reported to show positive correlation with the proliferation and metastasis of tumor, and STAT1 enhances innate and adaptive immunity, triggering in most instances anti-proliferative and pro-apoptotic responses in tumor cells (Avalle et al., 2012). Qing-Re-Huo-Xue (QRHX) formulae increases the expression of iNOS and decreases the expression of IL-6, TNF-α, and Arg-1 through the JAK2/STAT3 pathway, further reducing the numbers of TAMs and inhibiting tumor growth in lung cancer mouse model (Xu et al., 2017) (Figure 2 and Table 4). In the lung cancer mouse model, YPF also prolong the survival time of tumor mice through inhibiting the growth of lung cancer cells. In tumor tissues, the increased numbers of CD4+ T cells/macrophages are observed with the increased expression of IL-2 and IL-12 and decreased expression of TGF-β (Wang L. et al., 2019) (Table 4). In the 4T1 breast cancer mouse model, triptolide (TR), as one diterpenoid epoxide produced by *Tripterygium wilfordii* Hook. f (one TCM herb), was found to inhibit the expression of CD206, arginase 1, and CD204, and inhibit the secretion of anti-inflammatory cytokines, further inducing the decreased number of tumor-related M2 polarized macrophages to block tumor angiogenesis (Li et al., 2020) (Table 4).

#### Human Cells

For human lung cancer cell, Water extract of *Panax ginseng* C. A. Mey. and *Astragalus mongholicus* Bunge are revealed to reverse the polarity of TAMs from M2-like to M1-like by decreasing IL-10, TGF-β, Arg-1, and CD206 production on TAMs, consequently retarding the cancer invasion (Chen et al., 2019) (Figure 1 and Table 5). What is more, the reprogramming of TAMs toward M1-like macrophages is regulated by TCM Herbs, such as *Crocus sativus* L, which can elevate the expression of IL-1β and TNF-α to induce the development of a polarized phenotype of M1-like macrophages after tumor antigen stimulation, restoring their antigen presentation ability in human melanoma. These data indicate that *Crocus sativus* L has a special immunomodulatory effect (Shen et al., 2019) (Figure 1 and Table 5). Bu-Fei Decoction (BFD) inhibits the growth of both A549 and H1975 cell lines and reduces the expression of IL-10, PD-L1, and CD206 on TAM to restore their activity (Pang et al., 2017) (Figure 1 and Table 5). Recently, baicalein (BC) was found to regulate M2 polarization and inhibit the secretion of TGF-β1 to inhibit the growth and metastases of human breast cancer (Zhao X. et al., 2018) (Table 5). Therefore, CHM and their active components oppose the promotion effect of TAMs on tumor to inhibit the growth, invasion, and metastasis of tumor in TME.

**TABLE 5:** The roles of CHM on human TIMPs.

TCM herbs and their components	Human cells	TIMPs	Mechanisms	References
**WEPGAM**	A549 lung cancer cells	TAMs	To promote the transformation of M2 phenotype to M1 phenotype	Chen et al. (2019)
**CSL**	A375 melanoma cells	TAMs	To diminish TAM to impede tumor growth	Shen et al. (2019)
**BFD**	A549/NCI-H1975 Lung cancer cells	TAMs	To decrease TAM production	Pang et al. (2017)
**BC**	MDA-MB-231 breast cancer cells	TAMs	To regulate the polarization and function of TAMs	Zhao Y. et al. (2018)
**PPS**	Human cervical cancer cells	TADCs	To activate DCs to present antigens to T cell	Wang et al. (2020a)

*BC, baicalein; BFD, Bu-Fei decoction; CSL, *Crocus sativus* L; DC, dendritic cells; MDSCs, myeloid-derived suppressor cells; PPS, *Pinellia pedatisecta* Schott; TADC, decoction tumor-associated dendritic cells; TAM, tumor-associated macrophage; WEPGAM, water extract of *Panax ginseng* C. A. Mey. and *Astragalus mongholicus* Bunge.*

### The Function of CHM and Their Ingredients on DCs

Dendritic cells are the principal APCs of the human body, which can efficiently ingest, process, and present antigens under physiological conditions. TME affects aggregation, maturation, and survival of DCs, and hampers the antigen presentation of DCs and sustains dysfunctional DCs to escape immune recognition, leading to the formation of tumor-associated DCs (TADCs), which exhibits a low ability to present antigen and facilitates T cells differentiating to Treg subtype, further impairing T cell-mediated antitumor activity (Giovanelli et al., 2019; Lee et al., 2020). Therefore, it is an effective way for antitumor immunotherapy to boost antigen presentation ability of DCs.

#### Murine Cancer Cell Lines/Models

**FIGURE 3:**
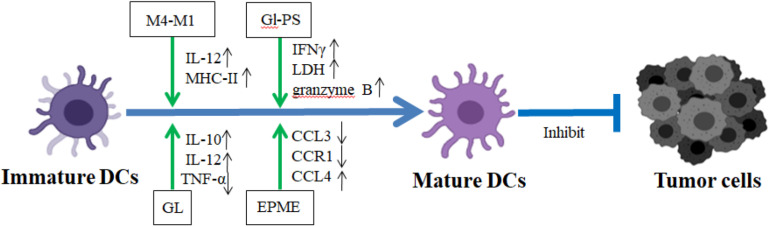
The regulatory mechanism of CHM on the maturation and function of DCs in TME. In TME, the tumor-associated DC (TADC) promoted the differentiation of T cells into Treg subtypes and further impairs the anti-tumor activity mediated by T cells. GL, Gl-PS, M4-M1, and EPME stimulated TADC maturation to bolster the antitumor activity. GL, glycyrrhizin; M4-M1, extracting M4 from protopanaxatriol and M1 from protopanaxadiol; Gl-PS, *Ganoderma lucidum* polysaccharides; EPME, *Echinacea purpurea* (L.) Moench extracts. Blue lines demonstrated the promotion (→) or inhibition (⊣) roles among immature dendritic cells, mature dendritic cells, and tumor cells. Green lines indicated the promotion (→) or inhibition (⊣) roles of CHM.

Chinese herbal medicine and their components play positive roles in the DC maturation stimuli. *Lycium barbarum* L. polysaccharide (LBP) was also found to play critical roles in DC maturation. LBP induces the functional maturation of murine DCs *in vitro* through the increased expression of Notch and Jagged and Notch targets Hes1 and Hes5. Additionally, the administration of LBP strengthens the cytotoxicity of DC-mediated CTLs on murine colon cancer cell CT26-WTCTLs (Wang W. et al., 2018) (Table 3). LBP also induces Toll-like receptor 2- and 4-mediated functional maturation of murine DCs via the activation of NF-κB (Zhu et al., 2013). *Ganoderma lucidum* polysaccharides (GLPS), one of the major categories of the bioactive ingredients of *Ganoderma lucidum*, exhibit multiple biological activities such as improvement of host immune function, prevention of oxidative damage, and inhibition of tumor with little toxicity (Cor et al., 2018) (Figure 3). Recent data demonstrated that GLPS stimulated DC maturation through the increased production of IFN-γ, further enhancing antitumor response of specific CTL on mast tumor cells (Cao and Lin, 2003) (Table 3). GLPS also elevates the co-expression levels of both CD11c and IA/IE on DC surfaces and augment protein production of IL-12 P40 on DCs (Cao and Lin, 2002) (Figure 3).

Our recent report has shown that Ginsenosides, as the functional contents of ginseng, enhance the antigen presentation function of DCs within the TME. Ginsenosides activate the activity of DCs and promote adaptive immune responses to exert anticancer effects in tumor-bearing mice (Zhang Y. et al., 2019). Both the purified glycyrrhizin (GL) and *Carthamus tinctorius* L. (CT) extract stimulate DC maturation to bolster antitumor activity. The former increases the production of IL-12 and IL-10 and decreased the production of TNF-α. The latter stimulates splenic T lymphocytes to secrete IFN-γ, significantly increasing the levels of TNF-α and IL-1β in tumor-bearing mice (Chang et al., 2011; Hua et al., 2012) (Figure 3 and Table 4). Wang Y. et al. (2019) found that *Pinellia pedatisecta* Schott (PPS) upregulated the expression of MHCII and CD80, CD86, and IL-12 on TADCs to promote the proliferation of CD4+ and CD8+ T cells in human cervical cancer, thereby eliciting further antitumor CTL responses (Table 4).

Dendritic cell vaccine is one newly emerging immunotherapeutic approach for the treatment and prevention of cancer, but major challenges remain particularly with respect to clinical efficacy. Polysaccharide components purified from *Astragalus mongholicus* Bunge or *Codonopsis pilosula* subsp. *pilosula* (PAMB PCPP) were displayed to induce the increased expression of mature DC markers, such as CD40, CD80, and CD86. It is an effective adjuvant to improve the metastasis efficiency of DC vaccine against 4T1 breast cancer in mice, indicating that those polysaccharides may contribute to the formulation of DC-based vaccine for cancer immunotherapy (Chang et al., 2015) (Table 4).

#### Human Cells

Most investigations are focused on the roles of CHM and its active components on murine DCs. Recently, their roles on the human DC maturation were also revealed. The effects of PPS on human TADCs were mediated through the inhibition of SOCS1 and activation of downstream JAK2-STAT1/STAT4/STAT5 pathways. Those data suggest that PPS is an effective immunomodulatory drug for antitumor treatment via the blockade of SOCS1 signaling in DCs (Wang et al., 2020a) (Table 5). Polysaccharide purified from *Ganoderma lucidum* (PS-G) increases the expression levels of IL-12p70, IL-12p35, CD80, CD83, CD86, and human leukocyte antigen (HLA)-DR on human monocyte-derived DC through NF-κB and p38 mitogen-activated protein kinase pathways, promoting the maturation of human monocyte-derived DCs (Lin et al., 2005). Extracting M4 from protopanaxatriol and M1 from protopanaxadiol (M4-M1) was shown to increase the expression levels of IL-12 on DCs to stimulate DC maturation in TME. In addition, M4-M1 increased the expression level of CD80, CD83, and CD86 on DCs to enhance the antitumor ability of T cell (Takei et al., 2004) (Figure 3). Echinacea (L.) Moench extract (EPME) downregulates the expression of CCL3, CCL8, CCR1, and CCR9 and upregulates the expression of CCL4 and CCL2 to trigger the maturation of human DCs (Wang et al., 2006) (Figure 3). Those results indicated that CHM promoted the maturation of both murine and human DCs, enhancing their present ability to tumor antigen efficiently in TME.

### The Effect of CHM and Their Elements on MDSCs From Murine Tumor Models

Myeloid-derived suppressor cells, as the important TIMPs, aggregate in TME and exhibit strong immunosuppressive activity to T cell antitumor response. In TME, plenty of IMCs were differentiated into large amounts of MDSCs, whereas the differentiation of MDSC into mature macrophage or DCs was prevented (Gabrilovich and Nagaraj, 2009). In the section, we discuss the functional roles of CHM on differentiation, expansion, and suppressive function of MDSCs within TME from murine tumor models, since the investigations about the regulation of CHM on tumor MDSCs are focused on murine tumor models mainly.

**FIGURE 4:**
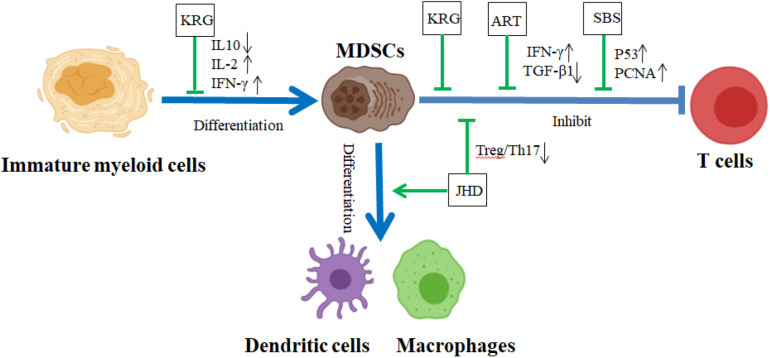
The effect of CHM on the differentiation and function of MDSCs in TME. In TME, MDSCs accumulated in TME and exhibited strong immunosuppressive activity against T cell antitumor response. KRG prevented the differentiation of IMCs into MDSCs with suppressive function, and JHD triggered the differentiation of MDSCs into mature DCs and macrophages. ART, KRG, SBS, and JHD impaired the suppressive function of MDSCs, further restraining the invasion and metastasis of tumor. ART, artemisinin; JHD, Jian-Pi-Hua-Yu decoction; KRG, Korean red ginseng; SBS, Shen-Ling-Bai-zhu San. Blue lines demonstrated the promotion (→) or inhibition (⊣) roles among immature myeloid cells, MDSCs, and T cells. Green lines indicated the promotion (→) or inhibition (⊣) roles of CHM.

Myeloid-derived suppressor cells are not present in the circulation under normal physiological conditions, but these cells accumulate in the tumor-bearing mice. MDSC accumulation was downregulated by TCM herbs, such as Shen-Ling-Bai-Zhu San (SBS) formula, Shu-Gan-Jian-Pi formula (SGJP), Water extract of Pilose Antler (PA), and RSV. In the colitis-associated colorectal cancer (CaCRC) mouse model, SBS upregulates β-catenin, p53, and proliferating cell nuclear antigen (PCNA), and reduces the mortality and the number of MDSCs. It also alleviates TGF-β1-induced EMT through downregulating N-cadherin (N-cad), Vimentin, Fibronectin, and Snail, and upregulating E-cadherin (E-cad) (Lin et al., 2015) (Figure 4 and Table 4). In breast cancer mouse models, both SGJP and PA inhibit the numbers of MDSCs to increase the proportion of CD4+ T cells, CD8+ T cells, and NK cells in peripheral blood of mice, further improving the survival rates of mice and blocking tumor growth (Lu et al., 2017; Zheng et al., 2018) (Table 4). In Lewis lung cancer-bearing mice, RSV was shown to diminish the accumulation of G-MDSCs and promote M-MDSC differentiation into macrophages and the expansion of CD8+IFN-γ+ cells (Zhao Y. et al., 2018) (Table 4). Recently, Yue et al. (2018) reported that four types of TCM herbs, *Andrographis paniculata* (Burm.f.) Nees (AP), *Eleutherococcus senticosus* (Rupr. & Maxim.) Maxim. (AS), *Camellia sinensis* Kuntze (CS), and *Scleromitrion diffusum* (Willd.) R.J. Wang (SD) reduced tumor tissue weights and tumor metastasis of both lung and liver, and decreased the numbers of both Tregs and MDSCs to coordinate the antitumor response of T cells to cancer cells, prolonging the survival period of mice in the metastatic breast cancer mouse model (Table 4). In the EL-4 thymoma mouse model, Korean red ginseng (KRG) was displayed to prevent the abnormal differentiation of IMCs into MDSCs and impair MDSC function, inducing T cell proliferation and secretion of both IL-2 and IFN-γ (Jeon et al., 2011) (Figure 4 and Table 4). In H22 hepatocellular carcinoma-bearing mice, Jianpi Huayu decoction (JHD) significantly diminishes the destruction of spleen structure and the ratios of between Treg and Th17, and increases the ratios of CTL, DC, and MDSCs in the spleen. JHD also promotes the differentiation of IMCs into macrophages and mDCs, and weakens the expression of ROS in MDSCs to impair the inhibitory effect of those MDSCs on CD4+ T cell proliferation (Xie et al., 2020) (Figure 4 and Table 4). In 4T1 breast cancer mouse model, which is a suitable experimental animal model for human mammary cancer, artemisinin (ART) significantly promotes 4T1 tumor cell apoptosis and decreases TGF-β levels and the numbers of both MDSC and Treg to inhibit tumor growth in mice (Cao Y. et al., 2019) (Figure 4 and Table 4).

The suppressive function of MDSC on T cell antitumor response has been studied broadly in our laboratory and other institutes (Qu et al., 2012; Fultang et al., 2019; Su et al., 2019). There are growing evidence that the immune-regulatory roles of CHM on the function of MDSCs become one of the major cancer immunotherapies of CHM progressively. CHM reverses the function of MDSCs through tumor-related signaling pathways such as the JAK/STATs and TGF-β/Smads pathway. Mao et al. (2018) found that, in breast cancer mouse models, Yang-He decoction (YHD) depressed the expression of iNOS, ARG-1, IL-6, TGF-β, and p-STAT3 on MDSCs and significantly increased the expression of IFN-γ and NKTs on CD4^+^T cells to shrink tumor growth (Table 4). Icariin (ICA) from *Epimedium sagittatum* (Siebold & Zucc.) Maxim downregulates the expression levels of IL-10, IL-6, S100A8/9, iNOS, and ROS on MDSCs to attenuate the roles of MDSCs through the inactivation of STAT3 (Zhou et al., 2011) (Table 4). In the murine sarcoma model, corosolic acid (CA) was revealed to induce the decreased expression levels of both cyclooxygenase-2 (Cox2) and CCL2 through the inactivation of Stat3 to impair the immunosuppressive activity of MDSCs (Horlad et al., 2013) (Table 4). Recently, Bao-Yuan-Jie-Du decoction (BYJD) is found to suppress the protein expression of TGF-β, Smad2, Smad3, p-Smad2/3, and Smad4 through the TGF-β/Smads signaling pathway to inhibit the recruitment of MDSCs in the lung and prolong the survival time of 4T1 tumor-bearing mice (Tian et al., 2020) (Table 4). In summary, CHM and their compounds stimulate the differentiation of MDSCs into mature myeloid cells, diminish the number and expansion of MDSCs, and restrain the suppressive function of MDSCs to block the tumor metastasis in TME.

## Conclusion

Chinese herbal medicine contains rich and diverse chemical components, including alkaloids, polysaccharides, glycosides, and flavonoids. These chemicals have a variety of biological functions. CHM plays an important role in inhibiting the tumor and mediating tumor TME. In the review, we focus on the impact of CHM on TIMPs within TME. CHM and their compounds induce the differentiation of TIMPs into mature or functional cells, promote the transformation of TAM from M2 type to M1 type, stimulate DC maturation, trigger the differentiation of MDSC into mature DC and macrophages, and weaken the inhibitory function of MDSCs, further inhibiting tumor invasion and metastasis in TME. Those evidences suggest that CHM and their active components may be regarded as one novel therapeutic method for cancer treatment.

## Future Prospect

Those CHM and their compounds may enhance the activity of other clinical antitumor antibodies such as anti-PD-L1 antibody on patients with cancer through inhibiting both the numbers and roles of TIMPs within TME. In addition, the therapeutic effects of multiple components from CHM on TIMPs may be examined and compared in different types of tumor to find the best candidates on tumor treatment. Those investigations may facilitate the clinical application of CHM on cancer immunotherapy.

## Author Contributions

PQ conceived and designed the work. ZL and ZQ coordinated technical support and funding. JL and PQ wrote the manuscript. YW, GL, XH, and HL acquired, analyzed, and interpreted the data. All authors read and approved the final manuscript.

## Conflict of Interest

The authors declare that the research was conducted in the absence of any commercial or financial relationships that could be construed as a potential conflict of interest.
